# A fresh look at polymicrobial bloodstream infection in cancer patients

**DOI:** 10.1371/journal.pone.0185768

**Published:** 2017-10-24

**Authors:** Cristina Royo-Cebrecos, Carlota Gudiol, Carmen Ardanuy, Helena Pomares, Mariona Calvo, Jordi Carratalà

**Affiliations:** 1 Department of Infectious Diseases, Hospital Universitari de Bellvitge, IDIBELL (Institut d´Investigació Biomèdica de Bellvitge), University of Barcelona, Barcelona, Spain; 2 REIPI (Spanish Network for Research in Infectious Diseases), ISCIII, Madrid, Spain; 3 Department of Microbiology, Hospital Universitari de Bellvitge, IDIBELL (Institut d´Investigació Biomèdica de Bellvitge), University of Barcelona, Barcelona, Spain; 4 CIBERes (CIBER de Enfermedades Respiratorias), ISCIII, Madrid, Spain; 5 Department of Hematology, Institut Català d’Oncologia, l’Hospitalet, University of Barcelona, Barcelona, Spain; 6 Department of Oncology, Institut Català d’Oncologia, l’Hospitalet, University of Barcelona, Barcelona, Spain; Seconda Universita degli Studi di Napoli, ITALY

## Abstract

**Objectives:**

To assess the current incidence, clinical features, risk factors, aetiology, antimicrobial resistance and outcomes of polymicrobial bloodstream infection (PBSI) in patients with cancer.

**Methods:**

All prospectively collected episodes of PBSI in hospitalised patients were compared with episodes of monomicrobial bloodstream infection (MBSI) between 2006 and 2015.

**Results:**

We identified 194 (10.2%) episodes of PBSI and 1702 MBSI (89.8%). The presence of cholangitis, biliary stenting, neutropenia, corticosteroids, neutropenic enterocolitis and other abdominal infections were identified as risk factors for PBSI. Overall, Gram-negative organisms were the most frequent aetiology, but *Enterococcus spp*. were especially frequent causes of Gram-positive PBSI (30.8%). Multidrug-resistant (MDR) organisms were more commonly found in PBSI than in MBSI (20.6% vs 12.9%; p = 0.003). Compared to patients with MBSI, those with PBSI presented with higher early (15% vs 1.4%; p = 0.04) and overall (32% vs 20.9%; p<0.001) case-fatality rates. Risk factors for overall case-fatality were a high-risk MASCC (Multinational Association of Supportive Care in Cancer) index score, corticosteroid use, persistent bacteraemia and septic shock.

**Conclusions:**

PBSI is a frequent complication in patients with cancer and is responsible for high mortality rates. Physicians should identify patients at risk for PBSI and provide empiric antibiotic therapy that covers the most frequent pathogens involved in these infections, including MDR strains.

## Introduction

Bloodstream infection (BSI) is a frequent complication in patients with cancer and results in important levels of morbidity and mortality [1]. Changes in the epidemiology of BSI have recently been documented in patients with cancer, showing a swift towards Gram-negative organisms as the leading cause of BSI in some institutions [2–4]. Also, infections due to MDR bacteria is an emerging problem in immunosuppressed patients with cancer, who are at higher for severe sepsis and poor outcomes than their immunocompromised peers [4–6].

Patients with cancer and chemotherapy-induced neutropenia, gastrointestinal mucositis, and medical devices in situ are at increased risk of BSI [7]. In this setting, BSI may be caused by multiple organisms in which the clinical presentation, microbiology and outcomes can vary from those caused by only one pathogen. The existing literature regarding such polymicrobial BSI (PBSI) is very limited, and mainly comprises old retrospective studies in the general population [8–12]. Few reports have specifically focused on patients with cancer [13–15]. Moreover, the lack of consistent PBSI definitions and the heterogeneity of populations in the previous studies makes it very difficult to understand the true relevance of PBSI [14].

In the recent years, changes in the general management of patients with cancer have occurred, including the introduction of newer types of myeloablative chemotherapies, transplants, and immunosuppressive agents, and changes in antibacterial and antifungal prophylaxis. These innovations may have influenced the frequency and characteristics of PBSI in patients with cancer.

The aim of this study was to explore the current incidence, clinical features, risk factors, aetiology, antimicrobial resistance and outcomes of PBSI in patients with cancer during the present era of widespread antimicrobial resistance.

## Materials and methods

### Setting, patients and study design

We conducted a prospective observational study at a 200-bed university referral centre for adults with cancer in Barcelona, Spain. We analysed all consecutive episodes of PBSI occurring in patients with cancer, including haematopoietic stem cell transplant recipients, from January 2006 to December 2015. Information on baseline characteristics, clinical features, aetiology, empirical antibiotic therapy and outcome were prospectively collected in a database, as part of the standard infectious disease management at our hospital. We also compared the characteristics of patients who died with those who survived to determine the factors influencing mortality. The study was approved by The Clinical Research Ethics Committee and Institutional Review Board of Hospital Universitari de Bellvitge.

### Definitions

PBSI was defined as present if two or more organisms were isolated from blood culture specimens collected from a patient during a period of <72h [13]. Chronic advanced cancer was considered in patients with confirmed metastatic disease (stage IV) and some stage III tumours (lung, pancreas, gastric, oesophagus, and urothelium) that were not suitable for treatment or were in progressive outbreak during treatment. Breast and prostate cancer with bone metastasis, colorectal cancer with resectable hepatic and lung metastasis, and metastatic germinal tumours were excluded.

BSI was considered nosocomial-acquired, healthcare-related or community-acquired, as previously described [16]. Neutropenia was defined as an absolute neutrophil count <500/mm^3^. Corticosteroid therapy was recorded if a patient was receiving corticosteroids at the time of BSI or at any point in the previous month. Shock was defined as a systolic blood pressure <90 mmHg that was unresponsive to fluid treatment or that required vasoactive drug therapy. Neutropenic enterocolitis was defined as the presence of fever, abdominal pain, and diarrhoea with imaging (ultrasonography, CT scanning) confirming the diagnosis [17]. The BSI was considered endogenous if no other sources were identified in neutropenic patient.

Initial empirical antibiotic therapy was considered inadequate if the treatment regimen did not include at least one antibiotic active *in vitro* against the infecting microorganism. Gram-negative bacilli were considered multidrug-resistant (MDR) if any of the following were present: a) extended-spectrum β-lactamase (ESBL)-producing *Enterobacteriaceae*; b) AmpC-cephalosporinase hyper-producing *Enterobacteriaceae*; c) carbapenem-resistant *Enterobacteriaceae*; d) microorganisms with intrinsic resistance mechanisms, such as *Stenotrophomonas maltophilia*; and e) MDR strains, including *Pseudomonas aeruginosa* and *Acinetobacter baumannii* [18].

The early case-fatality rate was defined as death within 7 days of the onset of BSI. The overall case-fatality rate was defined as death from any cause within the first 30 days of onset of BSI.

### Microbiologic studies

Two sets of two 8–10 mL blood samples (BactecPlus Aerobic and Anaerobic, BD) were taken 30 min apart from all patients who presented with fever ≥38°C or when BSI was suspected based on clinical signs or symptoms. Blood samples were processed in a BACTEC 9240 (from the year 2006 to May 2010) or a BACTEC-FX (since May 2010) apparatus (BD Microbiology Systems) with an incubation period of 5 days. Positive blood samples were sub-cultured onto chocolate agar.

Identification and antibiotic susceptibility testing of Gram-negative bacilli, *Enterococcus s*pp. and *Staphylococcus aureus* was performed using commercially available panels (MicroScan Beckman-Coulter). Identification of other *Streptococcus* spp. was performed by standard biochemical testing and antibiotic susceptibility with commercially available panels (Sensititre, TREK Diagnostic System). Anaerobe identification was performed by standard biochemical testing and antibiotic susceptibility by the E-test method (BioMérieux). In addition, identification has been performed by matrix-assisted laser desorption/ionisation(MALDI-TOF; Biotyper; Bruker Daltonics) since November 2012.The recommendations and criteria of the Clinical and Laboratory Standards Institute (CLSI) were used to define the susceptibility or resistance to antimicrobial agents [19]

### Statistical analysis

Continuous variables were compared using the Mann–Whitney *U* test and the Student *t*-test, as appropriate. Qualitative variables were compared using the chi-square test. Odds ratios (ORs) and 95% confidence intervals (CIs) were calculated, and a p-value of <0.05 was considered statistically significant. Multivariate conditional logistic-regression analysis of factors potentially associated with mortality was done that included all statistically significant variables in the univariate analysis, together with sex, age, and all clinically important variables, regardless of whether they were statistically significant. Kaplan–Meier curves were drawn to show the rate of survival in the PBSI and MBSI groups. The analysis was performed by stepwise logistic regression in SPSS version 17.0 (SPSS Institute Inc., Chicago, IL, USA).

## Results

### Patient characteristics

Of the 1896 episodes of BSI, we identified 194 (10.2%) episodes of PBSI in 179 patients and 1702 (89.8%) episodes of MBSI in 1155 patients. The baseline characteristics and clinical manifestations of patients with PBSI and MBSI are summarised in Table 1. Patients with PBSI were more likely to present with neutropenia and to have a biliary stenting in place. In addition, there was a trend towards the use of corticosteroids in this group of patients. The most frequent sources of BSI were endogenous (22.2%), catheter-related infection (19.8%) and urinary tract infection (10.6%). Cholangitis, neutropenic enterocolitis, abdominal infection and perirectal infection were more frequent in patients with PBSI, whereas urinary tract, respiratory tract and catheter infections were more common in patients with MBSI.

**Table 1:** Baseline characteristics and clinical presentation in patients with polymicrobial bloodstream infection and monomicrobial bloodstream infection.

Characteristic	PBSI N = 194(%)	MBSI N = 1702 (%)	*P* value
Age (years, median, range)	61 (14–90)	60 (21–84)	0.71
Male sex	122 (62.9)	1039 (61)	0.618
Haematological malignancy	116 (59.8)	1012 (59.5)	0.939
Haematopoietic stem cell transplant (HSCT)	26 (13.5)	288 (16.9)	0.261
Chronic advanced cancer	68 (39.3)	563 (38.7)	0.881
Other comorbidities	81 (41.8)	609 (35.8)	0.101
MASCC score < 21	45 (45.6)	291 (38.3)	0.173
**Neutropenia (<500 n/μL)**	**104 (53.6)**	**766 (45)**	**0.023**
Community acquired	16 (8.2)	179 (10.5)	0.324
Previous antibiotics (1 month)	96 (50)	822 (48.4)	0.682
Previous chemotherapy (1 month)	145 (74.7)	1195 (70.3)	0.193
Previous corticosteroid therapy (1 month)	83 (42.8)	609 (35.8)	0.056
Previous hospital admission (3mo)	100 (52.1)	842 (49.7)	0.527
**Biliary stenting**	**31 (16)**	**82 (4.8)**	<**0.001**
Other previous manipulations	18 (9.3)	146 (8.6)	0.746
**Source of BSI**			
Cholangitis	**39 (20.1)**	**149 (8.8)**	<**0.001**
Other abdominal site infections.	**26 (13.4)**	**138 (8.1)**	**0.013**
Neutropenic enterocolitis	**11 (5.7)**	**45 (2.6)**	**0.018**
Perirectal infection	**7 (3.6)**	**15 (0.9)**	**0.005**
Urinary tract	**5 (2.6)**	**197 (11.6)**	<**0.001**
Respiratory tract	**4 (2.1)**	**165 (9.7)**	<**0.001**
Endogenous source	52 (26.8)	369 (21.7)	0.104
**Catheter related**	**28 (14.4)**	**349 (20.5)**	**0.045**
Mucositis	3 (1.5)	58 (3.4)	0.2
Skin and soft tissue	2 (1)	50 (2.9)	0.162
Unknown	14 (7.2)	131 (7.7)	0.811
Fever ≥38°C	164 (84.5)	1426 (84.4)	0.969

### Risk factors for PBSI

Table 2 summarises the risk factors for PBSI by univariate and multivariate analysis. After adjustment, biliary stenting (OR 2.92; 95% CI, 1.52–5.61), neutropenia (OR 2.2; 95% CI, 1.44–3.35), corticosteroid therapy (OR 1.48; 95% CI, 1.08–2.03), cholangitis (OR 2.17; 95% CI 1.11–4.24) and abdominal infections (OR 2.48; 95% CI, 1.41–4.34) were identified as independent risk factors for PBSI. By contrast, urinary tract (OR 0.33; 95% CI, 0.13–0.85) and respiratory tract (OR 0.25; 95% CI, 0.09–0.71) infections were identified as low risk factors for PBSI.

**Table 2:** Risk factors for polymicrobial bloodstream infection by univariate and multivariate analysis.

Characteristics	PBSI n = 194 (%)	MBSI n = 1702 (%)	p-*Value*	Adjusted OR (95% CI)	p-*Value*
Sex (male)	122 (62.9)	1039 (61)	0.618	1 (.66–1.25)	0.825
Age (years, median, range)	61 (14–90)	60 (21–84)	0.71	1 (.99–1.02)	0.564
Biliary stenting	31 (16)	82 (4.8)	<.001	2.92 (1.52–5.61)	0.001
Neutropenia (<500/mm^3^)	104 (53.6)	766 (45)	0.023	2.2 (1.44–3.35)	0.001
Corticosteroid therapy	83 (42.8)	609 (35.8)	0.056	1.48 (1.08–2.03)	0.014
Cholangitis	39 (20.1)	149 (8.8)	<0.001	2.17 (1.11–4.24)	0.023
Other abdominal site	26 (13.4)	138 (8.1)	0.013	2.48 (1.41–4.34)	0.002
Neutropenic enterocolitis	11 (5.7)	45 (2.6)	0.018	1.88 (.93–3.82)	0.08
Perirectal infection	7 (3.6)	15 (.9)	0.005	3.87 (1.52–9.90)	0.005
Urinary tract	5 (2.6)	198 (11.6)	<.001	.33(.13-.85)	0.022
Respiratory tract	4 (2.1)	163 (9.6)	<.001	.25(.09-.71)	0.009
Catheter related	28 (14.4)	349 (20.5)	0.0431	.83(.52–1.32)	0.431

### Aetiology and antimicrobial resistance

Table 3 shows the aetiology of all episodes of BSI compared by groups. A total of 419 microorganisms were isolated in 194 episodes of PBSI. Among these, 27 episodes had 3 organisms and 2 episodes had 4 organisms. The most frequent combinations were Gram-positive plus Gram-negative organisms (36.1%) or Gram-negative plus Gram-negative organisms (30.4%).

**Table 3:** Aetiology of all episodes of bloodstream infection compared by groups.

Microorganisms	PBSI N = 419(%)	MBSI N = 1702 (%)
**Gram-negative**	**219 (52.52)**	**872 (51.23)**
* Escherichia coli*	74 (33.79)	421 (48.28)
* Pseudomonas aeruginosa*	43 (19.63)	124 (14.22)
* Klebsiella pneumoniae*	38 (17.35)	129 (14.79)
* Klebsiella oxytoca*	10 (4.57)	11 (1.26)
* Enterobacter spp*.	18 (8.22)	66 (7.57)
* Citrobacter spp*.	6 (2.74)	7 (0.8)
* Morganella morganii*	5 (2.28)	2 (0.23)
* Proteus spp*.	5 (2.28)	15 (1.72)
* Acinetobacter spp*.	3 (1.37)	6 (0.69)
* Salmonella enterica serovar enteritidis*	0	15 (1.72)
* Stenotrophomonas maltophilia*	1(0.46)	21 (2.41)
Other	16 (7.31)	55 (6.31)
**Gram-positive**	**173 (41.29)**	**735 (43.18)**
* Staphylococcus aureus*	15 (8.67)	115 (15.65)
Methicillin-resistant *S*. *aureus*	3 (1.73)	21 (2.86)
Coagulase-negative staphylococci	40 (23.12)	217 (29.52)
Viridans group streptococci	51 (29.48)	64 (8.71)
* Streptococcus gallolyticus*	6 (3.47)	17 (2.31)
* Streptococcus agalactiae*	2(1.16)	13(1.77)
* Streptococcus pneumoniae*	3 (1.73)	103(14.01)
* Enterococcus spp*.	53 (30.64)	130 (17.68)
* Enterococcus faecium*	25 (14.45)	78(10.61)
* Enterococcus faecalis*	18 (10.40)	44 (5.99)
* Other Enterococcus spp*.	10 (5.78)	8(1.09)
* Listeria monocytogenes*	1 (0.58)	25(3.40)
Other	2 (1.16)	30(4.08)
**Anaerobes**	**21 (5.04)**	**62 (3.64)**
* Clostridium spp*.	10 (50)	15 (24.19)
* Bacteroides spp*.	5 (23.8)	28 (45.16)
* Bacteroides fragilis*	2 (10)	21 (33.87)
Other	6 (30)	18 (29.03)
**Fungi**	**6 (1.44)**	**33 (1.94)**
* Candida albicans*	5 (83.3)	31 (93.94)
* Scedosporiumspp*.	1 (16.67)	0
* Fusarium solanii*	0	2 (6.06)
**Antibiotic Resistant**		
Multidrug-resistant (MDR)	40 (20.6)	219 (12.9)
Ampicillin-resistant vancomycin-susceptible *E*. *faecium*	22 (11.3)	68 (4)
ESBL-producing Enterobacteriaceae	11 (5.7)	75 (4.4)
AmpC-producing Enterobacteriaceae	4 (2.1)	19 (1.1)
MDR-*Pseudomonas aeruginosa*	1 (0.5)	12 (0.7)
Vancomycin-resistant *Enterococcus spp*.	6 (3.1)	3 (0.2)

Overall, Gram-negative organisms were the leading cause of BSI in both the PBSI and MBSI groups, with *Escherichia coli* and *Pseudomonas aeruginosa* being the most frequent causative agents. *Enterococcus spp*. were the most frequent Gram-positive organisms isolated in the cases of PBSI, followed by viridans group streptococci. In patients with MBSI, coagulase-negative staphylococci (CNS) were the most common Gram-positive agents, followed by *Enterococcus spp*. and *Staphylococcus aureus*. Among the anaerobes, clostridial infections were more common in polymicrobial episodes, whereas *Bacteroides* spp. were more frequently found in MBSI. Overall, infection due to MDR organisms was observed in 13.6% of cases, and it was more frequently found in episodes of PBSI (20.6% vs 12.9%; p = 0.003).

Considering only the polymicrobial episodes, patients with solid tumours presented more frequently with infection due to ESBL-producing Enterobacteriaceae compared to patients with haematological malignancies (11.5% vs 1.7%; p = 0.008).

### Antibiotic treatment and outcomes

Initial empirical antibiotic treatment and patients’ outcomes are detailed in Table 4. Patients with PBSI more frequently received carbapenems and a combination therapy for empirical antibiotic therapy than patients with MBSI. Among patients with PBSI, 54 were considered to have received inadequate initial empirical antibiotic therapy. Reasons for inappropriateness were as follows: 18 patients with *E*. *faecium* BSI received a β-lactam, 13 patients had MDR organisms, 10 patients did not receive any empirical treatment, 3 patients with fungal infection did not receive antifungals, and 3 patients with CNS infection were given cefepime and amikacin. The remaining 17 patients with PBSI received an antibiotic active against only one of the infective organisms.

**Table 4:** Therapeutic management and outcomes of polymicrobial bloodstream infection and monomicrobial bloodstream infection.

Characteristics	PBSI N = 194(%)	MBSI N = 1702(%)	*P* value
Empirical antibiotic treatment	184 (94.8)	1595 (93.5)	0.640
Combination therapy*	95 (51.6)	713 (44.7)	0.074
Monotherapy	89 (48.4)	882 (55.3)	0.074
* *β-lactam + β-lactamase inhibitor	44 (49.4)	405 (45.9)	0.526
Carbapenem	24 (27)	185 (21)	0.19
Cephalosporine	10 (11.2)	155 (17.6)	0.128
Aztreonam	1 (1.1)	11 (1.2)	1
Quinolone	3 (3.4)	56 (6.4)	0.261
Aminoglycoside	1 (1.1)	7 (0.8)	0.743
Glycopeptide	8 (9)	97 (11)	0.561
Inadequate empirical antibiotic therapy	54 (25.7)	438 (27.8)	0.527
Septic shock at presentation	24 (12.4)	191 (11.2)	0.64
Intensive care unit admission	19 (9.8)	126 (7.4)	0.24
Invasive mechanical ventilation	13 (6.7)	67 (4)	0.07
Early case-fatality rate (7d)	29 (15)	176 (10.4)	0.04
Overall case-fatality rate (30d)	62 (32)	349 (20.9)	<0.001

*More than 80% of the patients who received a combination therapy were treated with a β-lactam (mainly a cephalosporin or a carbapenem) plus an aminoglycoside.

**Fig 1:**
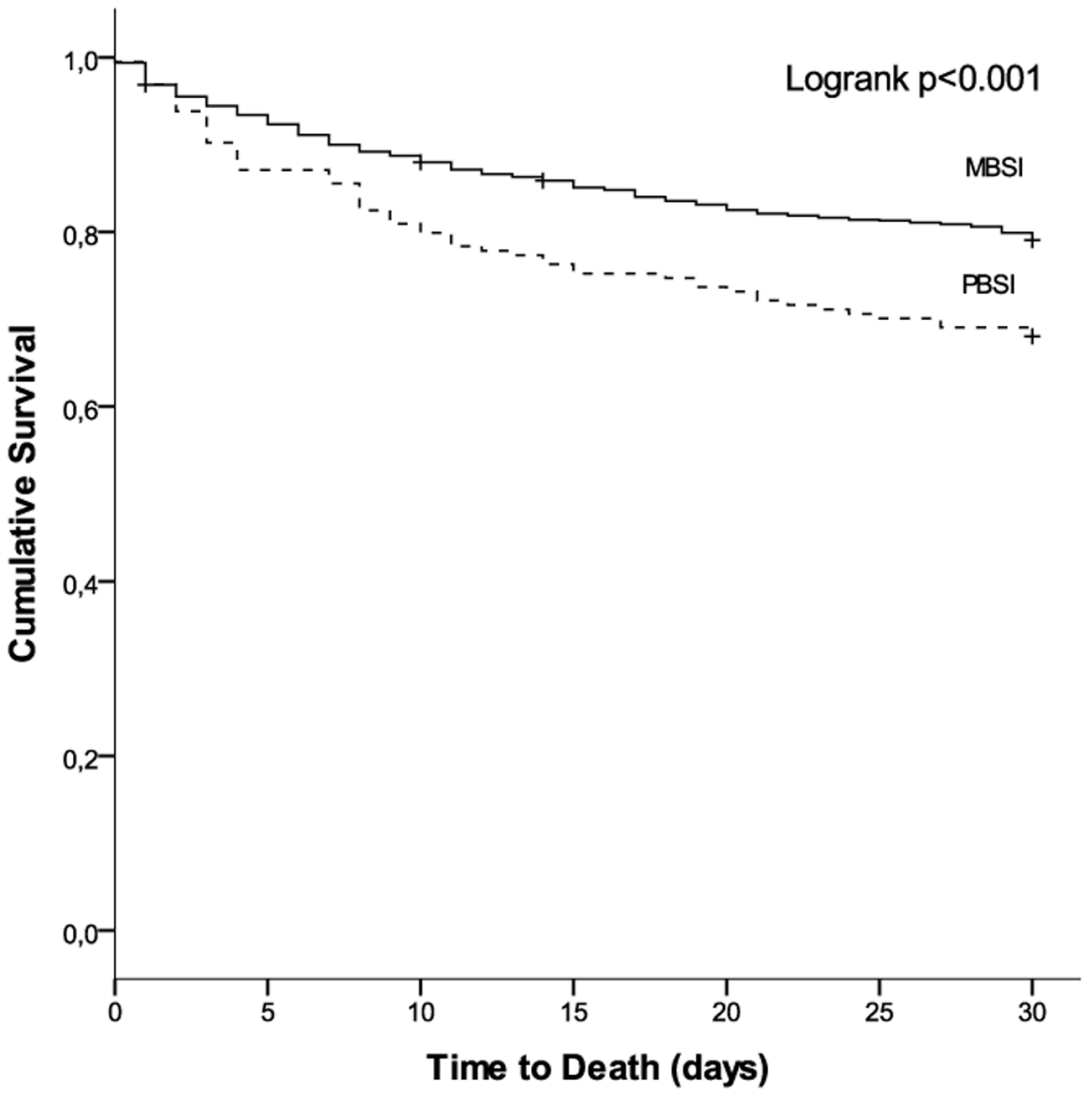
Kaplan–Meier survival curves of patients with polymicrobial bloodstream infection and monomicrobial bloodstream infection.

Patients with PBSI presented poorer outcomes with higher early and overall case-fatality rates than patients with MBSI. The Kaplan–Meier curves showing the rate of survival in the PBSI and MBSI groups are detailed in Fig 1. Among patients with PBSI, solid tumours were associated with a higher overall case-fatality rate than were haematological malignancies (39.7% vs 31%; p = 0.057), but there were no differences in early case-fatality rates (15.4% vs 14.8%; p = 0.909).

### Risk factors for overall case-fatality rate

Risk factors for the overall case-fatality rate are detailed in Table 5. After applying a logistic-regression model, the only variables found to be independent risk factors for overall case-fatality were a high-risk MASCC (Multinational Association of Supportive Care in Cancer) index score (OR 0.28; 95% CI, 0.17–0.46), corticosteroid therapy (OR 1.72; 95% CI, 1.1–2.7), persistent bacteraemia (OR 3.4; 95% CI, 1.82–6.33) and septic shock (OR 2.65; 95% CI, 1.51–4.66). PBSI was not a risk factor for overall mortality.

**Table 5:** Risk factors for overall case-fatality rate by univariate and multivariate analysis.

Characteristics	Survived n = 132 (%)	Died n = 62 (%)	p-*Value*	Adjusted OR (95% CI)	p-*Value*
Sex (male)	82 (62.1)	40 (64.5)	0.957	1.51 (.965–2.38)	0.071
Age (years, median, range)	60 (14–89)	63 (21–90)	<0.001	0.101 (.99–1.03)	0.148
Solid tumor	48 (36.4)	31 (50)	0.071	1.44 (.79–2.61)	0.226
MASCC risk score< 21*	26 (34.7)	19 (79.2)	<0.001	0.283 (.17-.46)	<0.001
Corticosteroid therapy	49 (37.1)	34 (54.8)	0.020	1.72 (1.1–2.7)	0.019
Persistent bacteremia	13 (10.2)	10 (23.3)	0.029	3.4 (1.82–6.33)	<0.001
Respiratory source	0	4 (6.5)	0.010	1.16 (0.48–2.79)	0.74
Septic Shock	11 (8.3)	13 (21)	0.013	2.65 (1.514–4.66)	0.001
Inadequate empirical antibiotic therapy	98 (74.2)	40 (65.5)	0.215		
Malignancy-related complications	27 (20.5)	25 (40.3)	0.004	1.87 (.52–6.74)	0.34
Multidrug-resistant bacteria	27 (20.5)	13 (21)	0.934		

*MASCC: Multinational Association for Supportive Care in Cancer

## Discussion

In this prospective study of a large cohort of patients with cancer, PBSI occurred in one out of ten patients. Multidrug resistance was frequent in those with PBSI, and outcomes were poorer, particularly in those with corticosteroid therapy, with a high-risk MASCC index score, and presenting with septic shock or persistent bacteraemia. The incidence of PBSI in the general population ranges from 6% to 14%; but it seems to increase in patients with cancer, reaching up to 32% in high-risk patients with leukaemia and in recipients of autologous haematopoietic stem cell transplants [8, 10– 12, 20, 21]. Although our results seem to be in line with these studies, it is difficult to assess the real incidence of PBSI over time because of the heterogeneity of the population studied and the lack of consistent definitions used [14].

We found that PBSI was more frequent in patients with perirectal infections, neutropenic enterocolitis or cholangitis, especially if they had a biliary prosthesis. As we have previously described, cholangitis is a frequent cause of PBSI in patients with solid tumours, and obstruction of the prosthesis may play a role in the pathogenesis of infection [22]. The abdomen was the most frequent source of PBSI, presumably because the site of entry of the infection is often the site of the primary tumour, and because of the high incidence of gastrointestinal ulcerations in patients with leukaemia [9, 13, 23].

As documented by other investigators, Gram-negative organisms were the most frequent causative agents in our study. Remarkably, enterococci were the most frequent Gram-positive pathogens in patients with PBSI. Compared to previous studies, in which streptococcal BSI was the predominant Gram-positive infection [13, 15], our data show that enterococci may be taking over from other Gram-positives. An interesting finding that has not previously been documented was the incidence of multidrug resistance, which reached 20% in our study. The most frequent MDR organisms were ampicillin-resistant vancomycin-susceptible *E*. *faecium*, followed by ESBL-producing Enterobacteriaceae. Infections caused by ampicillin-resistant *E*. *faecium* are increasing in patients with cancer, but their impacton outcomes seem to be less relevant than infections due to vancomycin-resistant strains [24, 25]. Moreover, there is still controversy regarding the association between *E*. *faecium* infection and mortality [24, 26, 27]. Likewise, infections due to ESBL-Enterobacteriaceae are also increasing in patients with cancer [28–32].

The emergence of multidrug resistance in patients with cancer is of special concern because inadequate initial empirical antibiotic therapy may negatively influence outcomes [30–31]. In this regard, we found that inadequate initial empirical antibiotic therapy was not associated with higher case-fatality rates. Nevertheless, it should be noted that infections in patients receiving inadequate therapy were mainly caused by *E*. *faecium* and CNS, which are intrinsically resistant to the most frequently used empirical antibiotic therapy in our institution (cefepime plus amikacin). As previously stated, it seems that ampicillin-resistant vancomycin-susceptible *E*. *faecium* BSI may not be clearly associated with poor outcomes. Moreover, BSI due to CNS has previously been identified as a predictor of lower mortality in neutropenic patients with haematological malignancies, as mentioned in other studies [7, 33,34].

We found lower case-fatality rates compared with other studies of PBSI involving immunocompetent and immunosuppressed cancer patients [8, 10, 11, 13]. However, it should be noted that most of the existing studies were performed several decades ago, and that the management of patients with PBSI has improved over the intervening period. Nevertheless, we found that a high-risk MASCC index score, corticosteroid therapy, persistent bacteraemia and septic shock were associated with higher mortality. The MASCC score is frequently used as a predictor of complications in patients with febrile neutropenia, with lower scores indicating a higher risk of developing severe complications and death[35]. Likewise, septic shock at presentation and the persistence of bacteraemia are clinical features associated with severe sepsis and/or uncontrolled sepsis [33]. Corticosteroid therapy to mitigate symptomatology has previously been identified as a risk factor for mortality in our cohort, mainly in those with advanced underlying malignancy [6, 24].This may be related to the fact that corticosteroids decrease the immune response and favour severe sepsis [36].

This study has some limitations that should be acknowledged. First, this is a single-centre study in a particular geographical area, so caution should be exercised when extrapolating the data to other settings. Second, as with any observational study, there is a potential for residual confounding; however, the strengths of the current study include the prospective collection of data, the large number of bacteraemia episodes in patients with cancer and the use of a uniform and comprehensive protocol for data collection.

In conclusion, PBSI is especially frequent in patients with cancer who have cholangitis, biliary stenting, or neutropenic enterocolitis. Enterococcal BSI is gaining increasing epidemiological importance, as is the emergence of multidrug resistance. Physicians should be able to identify patients at risk of PBSI and provide an initial empirical antibiotic regimen that covers the most frequent pathogens involved in this serious infection.

## References

[1] Gudiol C, Aguado JM, Carratalà J. Bloodstream infection in patients with solid tumors. Virulence. 2016; 7: 298–308. doi: 10.1080/21505594.2016.1141161 26787095 10.1080/21505594.2016.1141161 PMC4871686

[2] Gudiol C, Bodro M, Simonetti A, Tubau F, González-Barca E, Cisnal M, et al Changing aetiology, clinical features, antimicrobial resistance, and outcomes of bloodstream infection in neutropenic cancer patients. ClinMicrobiol Infect. 2013; 19:474–9. 10.1111/j.1469-0691.2012.03879.x 22524597

[3] Montassier E, Batard E, Gastinne T, Potel G, de La Cochetière MF. Recent changes in bacteremia in patients with cancer: a systematic review of epidemiology and antibiotic resistance. Eur J Clin Microbiol Infect Dis. 2013; 32: 841–50. doi: 10.1007/s10096-013-1819-7 23354675 10.1007/s10096-013-1819-7

[4] Mikulska M, Viscoli C, Orasch C, Livermore DM, Averbuch D, Cordonnier C, et al; Fourth European Conference on Infections in Leukemia Group (ECIL-4), a joint venture of EBMT, EORTC, ICHS, ELN and ESGICH/ESCMID. Aetiology and resistance in bacteraemias among adult and paediatric haematology and cancer patients. J Infect. 2014;68: 321–31. doi: 10.1016/j.jinf.2013.12.006 24370562 10.1016/j.jinf.2013.12.006

[5] Satlin MJ, Cohen N, Ma KC, Gedrimaite Z, Soave R, Askin G, et al Bacteremia due to carbapenem-resistant Enterobacteriaceae in neutropenic patients with hematologic malignancies. J Infec. 2016; 73: 336–45. 27404978 10.1016/j.jinf.2016.07.002 PMC5026910

[6] Gudiol C, Tubau F, Calatayud L, Garcia-Vidal C, Cisnal M, Sánchez-Ortega I, et al 2011. Bacteraemia due to multidrug-resistant Gram-negative bacilli in cancer patients: risk factors, antibiotic therapy and outcomes. J Antimicrob Chemother. 2011; 66: 657–63. doi: 10.1093/jac/dkq494 21193475 10.1093/jac/dkq494

[7] Klastersky J, Ameye L, Maertens J, Georgala A, Muanza F, Aoun M et al. Bacteraemia in febrile neutropenic cancer patients. Int J Antimicrob Agents. 2007; 30 Suppl 1:S51–9. 17689933 10.1016/j.ijantimicag.2007.06.012

[8] Bodey GP, Nies Ba, Freireich EJ. Multiple organism septicaemia in acute leukemia; Analysis of 54 episodes. Arch Intern Med. 1965; 116: 266–72. 14315659 10.1001/archinte.1965.03870020106019

[9] Hermans PE, Washington JA. Polymicrobial bacteremia. Ann Intern Med. 1970;73: 387–92. 4917179 10.7326/0003-4819-73-3-387

[10] Kiani D, Quinn EL, Burch KH, Madhavan T, Saravolatz LD, Neblett TR. The increasing importance of polymicrobial bacteremia. JAMA. 1979; 242: 1044–7. 470044

[11] Roselle GA, Watanakunakorn C. Polymicrobial bacteremia. JAMA. 1979;242: 2411–3. 40048

[12] Cooper GS, Havlir DS, Shlaes DM, Salata RA. Polymicrobial bacteremia in the late 1980s: predictors of outcome and review of the literature. Medicine (Baltimore). 1990;69: 114–23. 2181231

[13] Elting LS, Bodey GP, Fainstein V. Polymicrobial septicemia in the cancer patient. Medicine (Baltimore). 1986; 65: 218–25. 3724434 10.1097/00005792-198607000-00002

[14] Rolston KV, Bodey GP, Safdar A. Polymicrobial infection in patients with cancer: an underappreciated and underreported entity. Clin Infect Dis. 2007; 45: 228–33. doi: 10.1086/518873 17578784 10.1086/518873

[15] Trifilio S, Zhou Z, Fong JL, Zomas A, Liu D, Zhao C, et al Polymicrobial bacterial or fungal infections: incidence, spectrum of infection, risk factors, and clinical outcomes from a large hematopoietic stem cell transplant center. Transpl Infect Dis. 2015; 17: 267–74. doi: 10.1111/tid.12363 25648349 10.1111/tid.12363

[16] Friedman ND, Kaye KS, Stout JE, McGarry SA, Trivette SL, Briggs JP, et al Health care-associated bloodstream infections in adults: a reason to change the accepted definition of community-acquired infections. Ann Intern Med 2002; 137: 791–7. 12435215 10.7326/0003-4819-137-10-200211190-00007

[17] Gomez L, Martino R, Rolston KV. Neutropenic enterocolitis: spectrum of the disease and comparison of definite and possible cases. Clin Infect Dis. 1998; 27: 695–9. 9798018 10.1086/514946

[18] Magiorakos AP, Srinivasan A, Carey RB, Carmeli Y, Falagas ME, Giske CG, et al Multidrug-resistant, extensively drug-resistant and pandrug-resistant bacteria: an international expert proposal for interim standard definitions for acquired resistance. Clin Microbiol Infect 2012; 18: 268–81. doi: 10.1111/j.1469-0691.2011.03570.x 21793988 10.1111/j.1469-0691.2011.03570.x

[19] Clinical and Laboratory Standards Institute (CLSI). Performance Standards for Antimicrobial Susceptibility Testing: 26 th ed. CLSI supplement document M100-S26 (ISBN 1-56238-923-8 [Print]; ISBN 1-56238-924-6 [Electronic]). Clinical and Laboratory Standards Institute, 950 West Valley Road, Suite 2500, Wayne, Pennsylvania 19087 USA, 2016.

[20] Wisplinghoff H, Seifert H, Wenzel RP, Edmond MB. Current trends in the epidemiology of nosocomial bloodstream infections in patients with haematological malignancies and solid neoplasms in hospitals in the United States. Clin Infect Dis 2003; 36: 1103–10. doi: 10.1086/374339 12715303 10.1086/374339

[21] Haug JB, Harthug S, Kalager T, Digranes A, Solberg CO. Bloodstream infections at a Norwegian university hospital, 1974–1979 and 1988–1989: changing etiology, clinical features, and outcome. Clin Infect Dis. 1994; 19: 246–56. 7986895 10.1093/clinids/19.2.246

[22] Royo-Cebrecos C, Gudiol C, García J, Tubau F, Laporte J, Ardanuy C, et al Characteristics, aetiology, antimicrobial resistance and outcomes of bacteraemic cholangitis in patients with solid tumours: A prospective cohort study. J Infect. 2017;74: 172–8. doi: 10.1016/j.jinf.2016.10.008 27826062 10.1016/j.jinf.2016.10.008

[23] Reuben AG, Musher DM, Hamill RJ, Broucke I. Polymicrobial bacteremia: clinical and microbiologic patterns. Rev Infect Dis. 1989; 11: 161–83. 2649955 10.1093/clinids/11.2.161

[24] Gudiol C, Ayats J, Camoez M, Domínguez MÁ, García-Vidal C, Bodro M, et al Increase in bloodstream infection due to vancomycin-susceptible Enterococcus faecium in cancer patients: risk factors, molecular epidemiology and outcomes. PLoS One. 2013; 8:e74734 doi: 10.1371/journal.pone.0074734 24069339 10.1371/journal.pone.0074734 PMC3778008

[25] Kamboj M, Chung D, Seo SK, Pamer EG, Sepkowitz KA, Jakubowski AA, et al The changing epidemiology of vancomycin-resistant Enterococcus (VRE) bacteremia in allogeneic hematopoietic stem cell transplant (HSCT) recipients. Biol Blood Marrow Transplant. 2010; 16: 1576–81. doi: 10.1016/j.bbmt.2010.05.008 20685257 10.1016/j.bbmt.2010.05.008 PMC3670412

[26] Noskin GA, Peterson LR, Warren JR. Enterococcus faecium and Enterococcus faecalisbacteremia: acquisition and outcome. Clin Infect Dis. 1995; 20: 296–301. 7742433 10.1093/clinids/20.2.296

[27] DiazGranados CA, Zimmer SM, Klein M, Jernigan JA. Comparison of mortality associated with vancomycin-resistant and vancomycin-susceptible enterococcal bloodstream infections: a meta-analysis. Clin Infect Dis 2005; 41: 327–33. doi: 10.1086/430909 16007529 10.1086/430909

[28] Gudiol C, Calatayud L, Garcia-Vidal C, Lora-Tamayo J, Cisnal M, Duarte R, et al Bacteraemia due to extended-spectrum beta-lactamase-producing Escherichia coli (ESBL-EC) in cancer patients: clinical features, risk factors, molecular epidemiology and outcome. J AntimicrobChemother. 2010; 65: 333–41. 10.1093/jac/dkp411 19959544

[29] Kim SH, Kwon JC, Choi SM, Lee DG, Park SH, Choi JH, et al Escherichia coli and Klebsiella pneumonia bacteremia in patients with neutropenic fever: factors associated with extended-spectrum β-lactamase production and its impact on outcome. Ann Hematol 2013; 92: 533–541. doi: 10.1007/s00277-012-1631-y 23161391 10.1007/s00277-012-1631-y

[30] Tumbarello M, Sanguinetti M, Montuori E, Trecarichi EM, Posteraro B, Fiori B, et al Predictors of mortality in patients with bloodstream infections caused by extended-spectrum-beta-lactamase-producing Enterobacteriaceae: importance of inadequate initial antimicrobial treatment. Antimicrob Agents Chemother. 2007; 51: 1987–94. doi: 10.1128/AAC.01509-06 17387156 10.1128/AAC.01509-06 PMC1891412

[31] Ha YE, Kang CI, Cha MK, Park SY, Wi YM, Chung DR, et al Epidemiology and clinical outcomes of bloodstream infections caused by extended-spectrum-beta-lactamase-producing Escherichia coli in patients with cancer. Int J Antimicrob Agents 2013; 42: 403–9. doi: 10.1016/j.ijantimicag.2013.07.018 24071027 10.1016/j.ijantimicag.2013.07.018

[32] Trecarichi EM, Tumbarello M, Spanu T, Caira M, Fianchi L, Chiusolo P, et al Incidence and clinical impact of extended-spectrum-beta-lactamase (ESBL) production and fluoroquinolone resistance in bloodstream infections caused by Escherichia coli in patients with hematological malignancies. J Infect 2009; 58: 299–307. doi: 10.1016/j.jinf.2009.02.002 19272650 10.1016/j.jinf.2009.02.002

[33] Marín M, Gudiol C, Ardanuy C, Garcia-Vidal C, Jimenez L, Domingo-Domenech E, et al Factors influencing mortality in neutropenic patients with haematologic malignancies or solid tumours with bloodstream infection. Clin Microbiol Infect. 2015; 21:583–90. doi: 10.1016/j.cmi.2015.01.029 25680311 10.1016/j.cmi.2015.01.029

[34] Horasan ES, Ersoz G, Tombak A, Tiftik N, Kaya A. Bloodstream infections and mortality-related factors in febrile neutropenic cancer patients. Med Sci Monit. 2011;17(5):CR304–9. doi: 10.12659/MSM.881773 21525814 10.12659/MSM.881773 PMC3539578

[35] Klastersky J, Paesmans M, Rubenstein EB, Boyer M, Elting L, Feld R, et al The Multinational Association for Supportive Care in Cancer risk index: A multinational scoring system for identifying low-risk febrile neutropenic cancer patients. J Clin Oncol. 2000; 18: 3038–51. doi: 10.1200/JCO.2000.18.16.3038 10944139 10.1200/JCO.2000.18.16.3038

[36] Cooper M, Stewart P. Corticosteroid insufficiency in acutely ill patients. N Engl J Med 2003; 348: 727–34. doi: 10.1056/NEJMra020529 12594318 10.1056/NEJMra020529

